# Comprehensive surveillance of acute respiratory infections during the COVID-19 pandemic: a methodological approach using sentinel networks, Castilla y León, Spain, January 2020 to May 2022

**DOI:** 10.2807/1560-7917.ES.2023.28.21.2200638

**Published:** 2023-05-25

**Authors:** Tomás Vega-Alonso, Jose Eugenio Lozano-Alonso, Ana Ordax-Díez, on behalf of the VIGIRA Research Group

**Affiliations:** 1 Regional Public Health Directorate, Regional Health Ministry, Valladolid, Spain; 2 Members of the VIGIRA Research Group are listed under Acknowledgements

**Keywords:** Acute Respiratory Infections, Surveillance, Sentinel network, COVID-19, Influenza, respiratory virus

## Abstract

**Background:**

Since 1996, epidemiological surveillance of acute respiratory infections (ARI) in Spain has been limited to seasonal influenza, respiratory syncytial virus (RSV) and potential pandemic viruses**.** The COVID-19 pandemic provides opportunities to adapt existing systems for extended surveillance to capture a broader range of ARI.

**Aim:**

To describe how the Influenza Sentinel Surveillance System of Castilla y León, Spain was rapidly adapted in 2020 to comprehensive sentinel surveillance for ARI, including influenza and COVID-19.

**Methods:**

Using principles and methods of the health sentinel network, we integrated electronic medical record data from 68 basic surveillance units, covering 2.6% of the regional population between January 2020 to May 2022. We tested sentinel and non-sentinel samples sent weekly to the laboratory network for SARS-CoV-2, influenza viruses and other respiratory pathogens. The moving epidemic method (MEM) was used to calculate epidemic thresholds.

**Results:**

ARI incidence was estimated at 18,942 cases per 100,000 in 2020/21 and 45,223 in 2021/22, with similar seasonal fold increases by type of respiratory disease. Incidence of influenza-like illness was negligible in 2020/21 but a 5-week epidemic was detected by MEM in 2021/22. Epidemic thresholds for ARI and COVID-19 were estimated at 459.4 and 191.3 cases per 100,000 population, respectively. More than 5,000 samples were tested against a panel of respiratory viruses in 2021/22.

**Conclusion:**

Extracting data from electronic medical records reported by trained professionals, combined with a standardised microbiological information system, is a feasible and useful method to adapt influenza sentinel reports to comprehensive ARI surveillance in the post-COVID-19 era.

**Key public health message**

**What did you want to address in this study?**

Seasonal influenza has been monitored since 1996 by the Health Sentinel Network in Castilla y León, Spain. We wanted to examine the relevance and usefulness of expanding a comprehensive and integrated surveillance system to monitor a wider range of acute respiratory infections in the post-COVID-19 era.

**What have we learnt from this study?**

We showed that we could integrate large data sets from healthcare centres on patients with respiratory illness and laboratory results on the type of viral infection. We evaluated the usefulness of the results in the region of Castilla y León (Spain).

**What are the implications of your findings for public health?**

Our experience of building an enhanced system for surveillance of respiratory diseases could serve as a model for other national or regional surveillance systems for preparedness for influenza and other respiratory virus pandemics.

## Introduction

Since the mid-1990s, epidemiological surveillance of acute respiratory infections (ARI) in Spain has been limited to seasonal influenza, respiratory syncytial virus (RSV) and potential pandemic viruses [1]. At the international level, the World Health Organization (WHO) has maintained a global surveillance system for influenza viruses since 1947. Sentinel surveillance integrating epidemiological and virological information was established in Europe in 1996 through the European Influenza Surveillance Scheme [2] which, in 2008, was incorporated into the structure of the European Centre for Disease Prevention and Control (ECDC) as the European Influenza Surveillance Network [3]. Whereas the primary focus was on influenza-like illness (ILI), several European countries maintained complementary surveillance of all ARI (e.g. Belgium, France, Germany) while others estimated the incidence of ILI using virological information from samples of patients with non-specific ARI (e.g. Albania, Ukraine, Russia and Romania) [4,5]. In parallel, the European Reference Laboratory Network for Human Influenza (ERLI-Net) [6] was integrating data from virus culture, molecular diagnostic techniques and genetic sequencing.

Following outbreaks of avian influenza, severe acute respiratory syndrome (SARS), Middle East respiratory syndrome (MERS) and influenza A(H1N1)pdm09 that occurred between 2002 and 2010, surveillance of respiratory diseases has gained increasing attention on the agenda of national [7] and international public health institutions in order to estimate the risk and impact of a new influenza pandemic [8] and the effectiveness of influenza vaccination.

In 2018, the Red Centinela Sanitaria de Castilla y León (RCSCyL, Health Sentinel Network of Castilla y León) [9] conducted a pilot project for the surveillance of ARI [10], taking advantage of the sentinel methodology [11] and new information and communication tools and technologies (i.e. full electronic medical record (eMR) availability, database mining, improving of molecular diagnosis). As a result of this first experience, the Comprehensive Surveillance of ARI (VIGIRA) [12] programme was developed, which was launched in the first months of the COVID-19 pandemic and was operational during the 2020/21 and 2021/22 seasons.

Our aim was to describe how we transformed an influenza surveillance system to establish a comprehensive ARI surveillance system. We present the implemented methodology and the results obtained for the 2020/21 and 2021/22 influenza seasons in Castilla y León and discuss the suitability of the system.

## Methods

### VIGIRA surveillance system

The VIGIRA surveillance system is formed by a network of basic surveillance units (BSUs) in Castilla y León, a large autonomous community in the middle of Spain. Within each BSU, sentinel family doctors or paediatricians and sentinel nurses use the same dataset that stores clinical and epidemiological data on individuals with a unique identifier registered with each practice. As at 1 January 2022, the VIGIRA surveillance system contained 68 BSUs, 46 in family medicine and 22 in paediatric practices, which monitor around 62,000 people in total, representing 2.6% of the population of Castilla y León. The VIGIRA surveillance system also includes 14 microbiology laboratories run by the Public Health Service and the National Influenza Centre in Valladolid, Spain (NICV), which is a reference laboratory for influenza viruses.

### Reporting of acute respiratory infections 

The BSUs record the ARI in the eMR, which automatically codes a list of diseases based on International Classification of Diseases (ICD-10) codes previously used for ARI surveillance used by other authors [13] (Table 1). The BSUs used the WHO definition for the diagnosis of ARI [14] and the ECDC criteria [15] for ILI. A possible case of COVID-19 was considered in any person with sudden onset of at least one of the following symptoms: cough, fever or shortness of breath, according the ECDC surveillance case definition clinical criteria [16]. If no ARI-specific diagnosis was available, ICD-10 codes J06.0 to J06.9 were used. 

**Table 1:** Diseases included in the surveillance of acute respiratory infections, Castilla y León, Spain, January 2020−May 2022

International classification of diseases (ICD-10)^a^	Code
Acute tonsillitis, unspecified	J03.90
Acute bronchiolitis	J21
Acute bronchitis	J20.3 to J20.9
COVID-19	U07.1
Acute pharyngitis, unspecified	J02.9
Influenza due to other types of identified influenza virus	J10
Influenza with pneumonia due to other types of identified influenza virus	J10.0
Influenza due to identified influenza viruses	J09.X3, J09.X9
Influenza due to unidentified influenza virus	J11.00, J11.1
Unspecified acute lower respiratory tract infection	J22
Acute upper respiratory infections of multiple and unspecified sites	J06.0, J06.9
Acute obstructive laryngitis (croup) and epiglottitis	J05
Acute laryngitis and tracheitis	J04.0, J04.10, J04.2
Acute nasopharyngitis (common cold)	J00
Bacterial pneumonia, not classified elsewhere^a^	J15.0, A48.1, J15.9
Pneumonia due to SARS-associated coronavirus	J12.81
Pneumonia due to *Mycoplasma pneumoniae*^a^	J15.7
Pneumonia due to *Streptococcus pneumoniae*, pneumonia due to *Haemophilus influenzae*^a^	J13, J14
Viral pneumonia, not elsewhere classified	J12.0, J12.9
Bronchopneumonia, organism unspecified^a^	J18.0
Pneumonia, organism unspecified^a^	J18.9
Other specified respiratory disorders^b^	J98.8

SARS: severe acute respiratory syndrome.

^a^ Bacterial pneumonias included in this table describe community-acquired pneumonias, a high proportion of which are secondary to ARI of viral aetiology.

^b^ This code is included because the cases presented a clear seasonal trend.

A previously coded clinical diagnosis could be revised for patients to either COVID-19 (U07.1 and J12.81) or another of the ARI listed in Table 1, based on the laboratory test results of multiplex PCR analysis for severe acute respiratory syndrome coronavirus 2 (SARS-CoV-2). Diagnosis of otitis, sinusitis, epiglottitis and tonsillitis of presumed bacterial origin without symptoms of suspected ARI e.g. rhinitis, conjunctivitis, fever, malaise, pharyngitis and cough, were excluded. Unspecified diagnosis coded as fever, cough, dyspnoea and other non-specific symptoms were also not included. Reinfections in the same person were registered as a new incident case. Clinical and epidemiological data of the disease or patient were entered into the 'ARI Care Guide for the Sentinel Network', which was included in the eMR. 

### Sample collection and microbiological analysis

Each BSU collected samples of nasopharyngeal exudates from patients within 5 days of symptom onset. Each week, a maximum of two patients with ILI and two patients with another ARI of different ages and type of disease were selected for PCR analysis. A positive SARS-CoV-2 rapid antigen test did not preclude a second swab for confirmation and detection of other pathogens through the multiplex PCR analysis.

Our samples were submitted to the microbiology laboratories labelled as ‘Public Health Samples - Health Sentinel Network’. We ensured traceability of samples from sample collection, through laboratory processing, to referral some of the samples to the NICV and to the WHO Collaborating Centre for Reference and Research on Influenza at The Francis Crick Institute (London, United Kingdom) for complementary analysis.

Sentinel samples were analysed by multiplex PCR to detect the following respiratory pathogens: adenovirus, bocavirus, coronaviruses 229E, HKU1, NL63 and OC43, human enterovirus, influenza A, influenza A(H1), influenza A(H1)2009, influenza A(H3), influenza B (Victoria and Yamagata lineages), MERS-CoV, SARS-CoV-2, human metapneumovirus, parainfluenza virus 1, 2, 3 and 4, human rhinovirus and RSV A and B. The FilmArray multiplex PCR system (Biomérieux) was used in 13 of the 14 microbiology laboratories and the Allplex Respiratory Panel Assays (Seegene Inc.) was used in one. In the NICV laboratory, the Luminex NxTAG Respiratory Panel (Luminex Corporation) was used.

Influenza-positive samples from the microbiology laboratories were submitted to the NICV for culture and analysis by end-point RT-PCR, and B lineages were studied through specific analysis. Each sample, test type and results based on Logical Observation Identifier Names and Codes (LOINC) [17] were recorded in a web-based application and stored in a database accessible for statistical analysis.

In addition to sentinel samples, the microbiology laboratories reported positive cases of influenza, RSV and, to a lesser extent, other respiratory viruses from non-sentinel samples. These non-sentinel samples were collected in ARI hospitalised patients or in the hospital emergency rooms.

### Data integration and data analysis

The disease codes listed in Table 1 were downloaded weekly, on Tuesdays, with the cases diagnosed in the n week (finishing on the Sunday prior) as well as the updated cases of the n−1 week. Patients registered with each practice were checked and duplicates were removed. A duplicate was defined as any ARI with the same patient identifier and onset date interval less than 7 days, regardless of a different code. ARI in the same person with onset date interval more than 7 days were considered reinfections. If a duplicate was detected, our algorithm assigned the following priority: (i) COVID-19 with a virological diagnosis and (ii) the first occurrence regardless of the type of ARI. The unique individual identifier and the date of initiation of the disease were used to link the virological information. The weekly monitored population was the sum of the populations of active BSUs per week.

The impact of the pandemic on the age-adjusted weekly incidence per 100,000 population of total ARI, ILI and COVID-19 cases from week 1 2020 to week 20 2022 is described. Age-adjusted cumulative incidence rates per 100,000 population for each disease and specific rates by age group were estimated for the 2020/21 (from week 21 2020 to week 20 2021) and 2021/22 (from week 21 2021 to 20 2022) seasons.

Using the features of the moving epidemic method (MEM) [4,18], a new approach was used to calculate the number of waves of ARI and COVID-19 during the entire study period and to estimate the respective epidemic thresholds, assuming a minimum weekly cumulative rate increase of 3% and a minimum wave representation of 1.5%. The epidemic thresholds were calculated with a windows parameter of 3.5 using the Moving Epidemic Method Shiny Web Application V2.15. Pearson correlation coefficient was used to compare the VIGIRA COVID-19 series with the universal surveillance series in Castilla y León for the period of the study before week 12 2022, when national surveillance strategy was limited to patients aged 65 and more, and separately for epidemic and non-epidemic periods. Finally, the respiratory pathogens detected per week in the 2021/22 season were described.

## Results 

**Figure 1:**
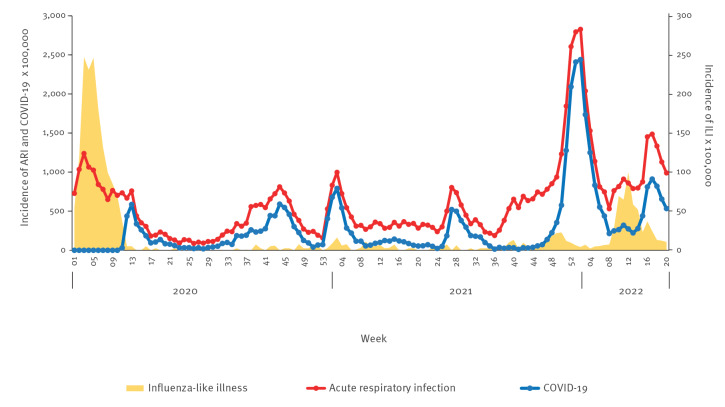
Weekly incidence rates per 100,000 population of acute respiratory infections (n = 53,238), COVID-19 (n = 20,642) and influenza-like illness (n = 1,733), adjusted by age, Castilla y León, Spain, week 1 2020–week 20 2022

ARI: acute respiratory infection; ILI: influenza-like illness.

**Figure 2:**
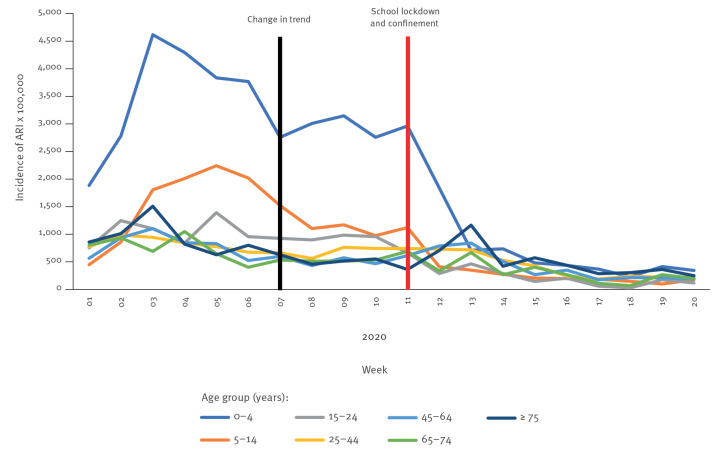
Weekly incidence rates of acute respiratory infections per 100,000 population by age group at the beginning of the COVID-19 pandemic, Castilla y León, Spain, week 1 2020–week 20 2020 (n = 7,527 infections)

As expected for a seasonal ARI and ILI wave, a seasonal 2019/20 peak was followed by a downward trend (observed at the beginning of the timeframe in Figure 1). At week 8 2020, while ILI declined unexpectedly sharply, ARI rates plateaued at above 700 cases per 100,000 population. This plateau, observed across nearly all age groups since week 7 2020, was most evident in children under 15 years of age (Figure 2). ARI rates remained stable until the school closure and population confinement were put into place on 9 March and 14 March, corresponding to week 11 2020. During week 14, the rates of ARI rapidly declined.

ARI: acute respiratory infection.

Change in trend: week in which the downward trend ceased.

### Incidence of acute respiratory infections, COVID-19 and influenza-like illness by season

The incidence of ARI in Castilla y León was 18,942 cases per 100,000 population in 2020/21 and 45,223 in the 2021/22 season (Table 2). COVID-19 was the primary cause of this observed increase, as the incidence rose from 9,909 to 23,573 cases from the first to the second season. All other ARI also show a 2–3-fold increase, except ILI, which increased by a factor of 4.8.

**Table 2:** Adjusted incidence rate of acute respiratory infections per 100,000 population by season and type of disease, Castilla y León, Spain, seasons 2020/21 (n = 12,103 cases) and 2021/22 (n = 33,608 cases)

Disease	Season		Fold increase
	2020/21	2021/22	
All ARI	18,942	45,223	2.4
Acute nasopharyngitis (common cold)	2,164	5,314	2.5
Acute pharyngitis	1,684	3,822	2.3
Acute tonsillitis	662	1,402	2.1
Acute laryngitis and tracheitis	280	745	2.7
Acute upper respiratory infections of multiple and unspecified sites	2,362	5,452	2.3
Acute bronchitis and bronchiolitis	239	795	3.3
Pneumonias and bronchopneumonias	181	229	1.3
COVID-19 or COVID-19 pneumonia	9,909	23,573	2.4
Influenza caused by specified or unspecified influenza viruses	151	729	4.8
Other respiratory diseases	1,310	3,162	2.4

Rates have been adjusted for the Castilla y León population age groups.

The incidence of ARI in children under the age of 5 years was 79,001 per 100,000 population in the 2020/21 season and 177,138 in the 2021/22 season. COVID-19 cases rose from 12,559 to 29,085 per 100,000 population in children under 5 years, but the increase was less pronounced in those aged over 75 years, where cases rose from 9,177 to 16,806 per 100,000 population (Table 3). The incidence of ILI was negligible in the 2020/21 season but exceeded 1,000 cases per 100,000 population in those less than 25 years of age in the 2021/22 season.

**Table 3:** Cumulative rates of acute respiratory infections per 100,000 population by disease and age, Castilla y León, Spain, seasons 2020/21 (n = 12,103 cases) and 2021/22 (n = 33,608 cases)

Disease	Season													
	2020/21							2021/22						
Age group (years)	0–4	5–14	15–24	25–44	45–64	65–74	≥ 75	0–4	5–14	15–24	25–44	45–64	65–74	≥ 75
All ARI	79,001	31,557	24,220	17,316	13,625	11,773	15,750	177,138	62,235	65,050	46,515	33,615	29,635	30,980
Acute nasopharyngitis (common cold)	23,477	6,501	1,428	1,379	853	756	856	41,221	9,025	6,079	4,291	3,218	3,108	3,100
Acute pharyngitis	3,504	3,191	2,277	1,670	1,269	1,270	1,297	8,506	5,771	7,147	3,731	2,830	3,371	2,310
Acute tonsillitis	2,965	1,182	2,156	846	233	89	56	6,528	2,140	5,516	1,643	377	299	82
Acute laryngitis and tracheitis	5,289	713	0	67	74	22	134	12,181	2,045	195	321	220	278	114
Acute upper respiratory infections of multiple and unspecified sites	23,301	6,062	2,605	1,607	986	938	1,034	46,656	9,696	7,612	3,958	2,926	3,012	2,836
Acute bronchitis and bronchiolitis	1,560	342	90	101	149	156	494	10,912	510	190	407	428	541	835
Pneumonias and bronchopneumonias	156	20	58	56	133	311	550	911	68	48	42	178	174	766
COVID-19 disease or COVID-19 pneumonia	12,559	11,545	14,296	10,389	8,948	7,250	9,177	29,085	28,359	33,098	28,861	21,165	15,593	16,806
Influenza caused by specified or unspecified influenza viruses	208	117	234	158	209	90	19	2,325	1,196	1,417	863	583	283	145
Other respiratory diseases	5,982	1,884	1,076	1,043	771	891	2,133	18,813	3,425	3,748	2,398	1,690	2,976	3,986

The MEM approach detected six ARI waves during the study timeframe (Figure 1). The first ARI wave included the first wave of COVID-19 and lasted until week 14 2020. The second wave appeared during weeks 36–48 in 2020, which was immediately followed by the third wave coinciding with predominantly SARS-CoV-2 Alpha (Phylogenetic Assignment of Named Global Outbreak (Pango) lineage designation B.1.1.7) variant and sublineages circulation, until week 7 2021. A plateau period with a slight increase in COVID-19 cases was followed by the fourth ARI wave from week 24 to 35 2021, during predominantly Delta (Pango lineage designation B.1.617) variant and sublineages circulation. The fifth and most intense ARI wave started in week 49 2021 and included the fifth COVID-19 wave, when predominantly Omicron (Pango lineage designation BA.1) variant and sublineages circulated. Finally, a bimodal sixth wave of ARI included the influenza epidemic from weeks 10–14 2022 and the sixth wave of COVID-19, again predominantly Omicron (Pango lineage designation BA.2) variant and sublineages circulation.

**Figure 3:**
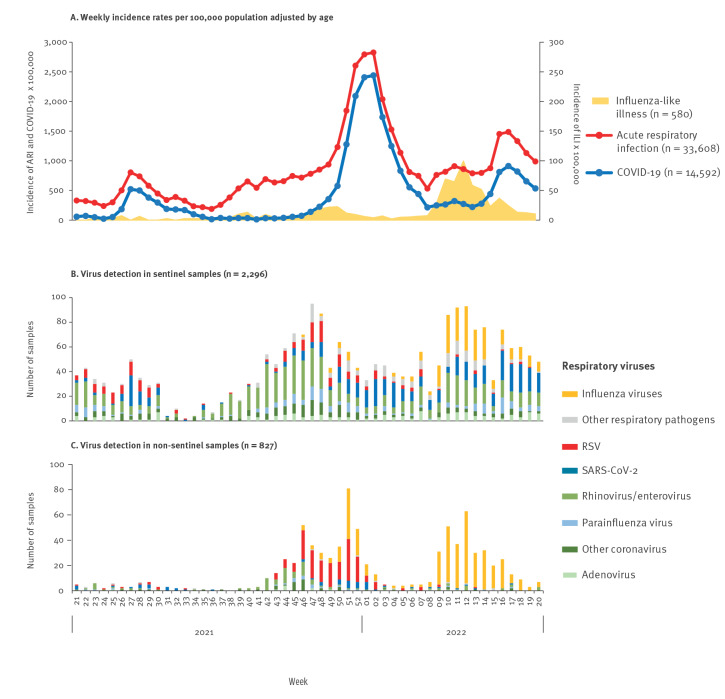
Weekly incidence rates of acute respiratory infections, COVID-19 and influenza-like illness per 100,000 population adjusted by age, and weekly number of respiratory viruses detected in sentinel and non-sentinel samples, Castilla y León, Spain, 2021/22 season

ARI: acute respiratory infection; RSV: respiratory syncytial virus; SARS-CoV-2: severe acute respiratory syndrome coronavirus 2.

These six waves caused by SARS-CoV-2 were the major contributors to ARI in both seasons since the beginning of the COVID-19 pandemic. The fifth wave of ARI started 10 weeks ahead of the fifth wave of COVID-19; this was in part caused by an increased circulation of rhinovirus/enterovirus and RSV (Figures 3B and 3C). The MEM epidemic thresholds for ARI and COVID-19 were estimated at 459.4 and 191.3 per 100,000 population, respectively; the fifth wave was excluded because of its intensity and abnormal duration.

The ILI series shows the epidemic of the 2019/20 season that occurred between January and February 2020, and two negligible waves in the winters of 2020 and 2021 (Figure 1). In week 10 2022, ILI exceeded the MEM epidemic threshold for the first time since the start of the COVID-19 pandemic, resulting in an epidemic of low intensity and short duration of only 5 weeks (Figures 1 and 3).

### Sentinel estimates and universal notification consistency for COVID-19

The weekly incidence estimates for COVID-19 are consistent with the universal surveillance [19] conducted in Castilla y León until the change of national strategy (week 12 2022). The combined Pearson correlation coefficient before that week was 0.997 (p < 0.001), and 0.996 (p < 0.001) and 0.921 (< 0.001) for the epidemic and non-epidemic periods, respectively.

### Virological surveillance in the 2021/22 season

In the 2,840 sentinel samples, co-circulation of SARS-CoV-2 and RSV was observed in the summer period coinciding with the fourth wave of COVID-19 (Figure 3). This was followed by an increase in rhinoviruses, enteroviruses, parainfluenza viruses and other coronaviruses, which accounted for up to 60% of all detections in weeks 37–50 2021, and coincided with an increase in ARI from week 37 2021 onwards. The fifth wave of COVID-19 emerged from week 51 2021 onwards and was correlated with detection of SARS-CoV-2, accounting for 20–50% of detections until week 7 2022.

In the 2,206 non-sentinel samples, we detected an increasing circulation of influenza A(H3) viruses between weeks 49–52 2021, which coincided with a small wave of influenza in primary care. A second wave of influenza A(H3) virus started in week 9 2022, corresponding to a substantial increase in ILI in the community for the 5 weeks following. RSV overtook influenza and was circulating between weeks 45–52 2021.

Starting in week 14 2022, rhinovirus, influenza viruses and SARS-CoV-2 activity was continuously detected and led to the sixth wave of COVID-19, which lasted until the end of the study period.

## Discussion

The incidence of ARI in Castilla y León was affected by the COVID-19 pandemic, with a drop in the 2020/21 season (18,942 cases per 100,000 population) and a large rise in the 2021/22 season (45,223 cases per 100,000 population), whereas the average in the 10 previous seasons was estimated in 28,000 cases per 100,000 population [10].

The estimated ARI in the 2020/21 season increased by a factor of 2.4 in the 2021/22 season. This increase in ARI, observed for all types of conditions except influenza and pneumonias, suggests an increased circulation of the group of respiratory viruses that was controlled by non-pharmacological interventions (NPIs), e.g. travel restrictions and quarantine upon arrival, social distancing, school and workplace closures, mask wearing, surface disinfection and enhanced hand hygiene, which proved effective in the 2020/21 season [20,21].

The six COVID-19 waves differ in duration and intensity, likely because of the combined impact of the intrinsic transmissibility and immune escape of new SARS-CoV-2 variants, the changes in the NPIs and the impact of the vaccination campaigns.

ILI cases disappeared in the 2020/21 season after the steep decline of ARI, which occurred following the lockdown in response to rising COVID-19 cases in March 2020. In the 2021/22 season, ARI cases increased above the epidemic threshold, mainly affecting the paediatric population. A previous study showed a decrease in influenza A and B laboratory detections concurrent with NPI efforts to control COVID-19 transmission in Canada [22].

Between weeks 7–11 2020, a change in the decreasing trend of ARI caused by early circulation of SARS-CoV-2 was observed; retrospective serological results confirm the detection of SARS-CoV-2 in ARI patients on 11 and 13 February (week 7 2020) [23].

In the Castilla y León community, sentinel surveillance has proven to be useful for ARI surveillance [14,24,25]. Our findings suggest that a population between 30,000 and 60,000 people, representative of a territory, ensures adequate accuracy for epidemic thresholds above 50 cases per 100,000 population with high diagnostic sensitivity and specificity, as has been discussed previously [26-29]. Our study shows a quite accurate correlation of COVID-19 sentinel incidence estimates with the registration of cases in the whole population.

The new paradigm of epidemiological surveillance is based on integrated, complete, continuous, timely, specific and quality population-based information that can be extracted from population-based large datasets and reported by professionals, who are knowledgeable about the objectives of the surveillance, and combined with the diagnostic specificity provided by the laboratory. Sentinel networks, which are based on representativeness, voluntariness, flexibility, national and international comparability, integration of epidemiological and virological data, feedback, training and recognition, are suitable tools for population-based surveillance of ARI and early detection of emerging and potentially pandemic pathogens, as well as for comparison of intensities between seasons or between countries [4,14,18]. The detection of outbreaks or infrequent variants, which is a limitation of sentinel sample surveillance, would need a different approach, such as the severe acute respiratory infections registry or the implementation of surveillance systems in care homes. On the contrary, advantages of the sentinel sample approach are the access to the trajectory of the cases and the sustainability of the surveillance system.

Universal surveillance of all ARI requires at least the separation of ILI. Any further differentiation of respiratory syndromes will require training, experience and a standardised coding system that can be achieved with a sentinel system. Virological surveillance brings specificity and interpretability to the observations. Unfortunately, we could not describe the virus circulation in the 2020/21 season because our virological surveillance system was in a pilot phase with partial data and without a formal validation, which is a limitation to understanding which pathogens were circulating at the beginning of the COVID-19 pandemic. In our series, between weeks 37–46 2021, the increase in ARI before the fifth wave of COVID-19 was a consequence of intense circulation of rhinovirus/enterovirus, RSV, parainfluenza and other coronaviruses. Likewise, the presumed COVID-19 wave around week 14 2021 was associated with a high incidence of other ARI, caused by high circulation of other respiratory pathogens not described in this article, predominantly rhinovirus/enterovirus, parainfluenza, but also adenovirus and other coronaviruses. Taken together, syndromic surveillance does not contribute substantially to the detection and control of emerging ARI or those with pandemic risk potential, if not accompanied by comprehensive laboratory surveillance of circulating respiratory viruses.

The selection of patients for swab sampling is also a matter of controversy. Systematic sampling would require more weekly samples to detect viruses of special interest (such as influenza or RSV) at the beginning and end of epidemics. As for COVID-19, the sensitivity to detect variants circulating at low levels is a priority [30] that is difficult to achieve with a sentinel system and systematic or random sampling in patients with ARI. One possible solution could be to perform a complementary selective sampling by type of disease and clinical and epidemiological characteristics, i.e. vaccinated cases, reinfections or immunocompromised cases, in order to maintain the representativity of the circulating viruses through the systematic sampling.

Non-sentinel virological information has proven to be very useful. The discordance between ILI rates and sentinel and non-sentinel detections of influenza virus could be explained by the reduction of primary care consultations and the increased number of patients going directly to the hospital emergency services during the pandemic period.

The need to implement a national surveillance system for ARI at national and European levels (ECDC, WHO/Europe) seems unquestionable. A recent article, which describes the adaptation of the primary care influenza sentinel surveillance systems for COVID-19 surveillance in seven European countries concluded that more challenges and experiences will help us make better and more informed decisions on how to integrate sentinel surveillance for influenza viruses and other respiratory pathogens with sentinel surveillance of SARS-CoV-2 [31]. In Spain, the National Health System has common elements in all Health and Surveillance Systems of the Autonomous Communities that can be used in the development of standardised methodologies and common indicators for intra- and international comparison. The principles of the development of VIGIRA are the strict application of the sentinel methodology, the inclusion of all ARI without breaking the historical series of influenza data, and the balance of quality, completeness and specificity of the diseases under surveillance. In addition, the international surveillance standards and operational considerations for respiratory virus surveillance of the ECDC and WHO are maintained [32], and the active participation of sentinel professionals and microbiology laboratories is encouraged.

## Conclusions

Respiratory viruses represent an important public health concern because of the high morbidity and mortality, the economic and social impact, and the risk of a pandemic. VIGIRA will need a formal evaluation in the coming season, but could be an example of valid, timely, comprehensive, comparable and sustainable surveillance system useful in preparedness and response of these threats worldwide.
